# Genet assignment and population structure analysis in a clonal forest-floor herb, *Cardamine leucantha*, using RAD-seq

**DOI:** 10.1093/aobpla/plz080

**Published:** 2019-12-20

**Authors:** Michiaki Tsujimoto, Kiwako S Araki, Mie N Honjo, Masaki Yasugi, Atsushi J Nagano, Satoru Akama, Masaomi Hatakeyama, Rie Shimizu-Inatsugi, Jun Sese, Kentaro K Shimizu, Hiroshi Kudoh

**Affiliations:** 1 Center for Ecological Research, Kyoto University, Hirano Otsu, Japan; 2 Faculty of Life Sciences, Ritsumeikan University, Nojihigashi, Kusatsu, Japan; 3 Faculty of Engineering, Utsunomiya University, Yoto, Utsunomiya, Japan; 4 Faculty of Agriculture, Ryukoku University, Yokatani, Seta Ohe-cho, Otsu, Japan; 5 National Institute of Advanced Industrial Science and Technology (AIST), Aomi, Koto-ku, Tokyo, Japan; 6 Department of Evolutionary Biology and Environmental Studies, University of Zurich, Winterthurerstrasse, Zurich, Switzerland; 7 Functional Genomics Center Zurich, Winterthurerstrasse, Zurich, Switzerland; 8 Humanome Lab., Inc. 2-4-10-2F, Tsukiji, Chuo-ku, Tokyo, Japan; 9 Kihara Institute for Biological Research, Yokohama City University, Maioka, Totsuka-ku, Yokohama, Japan

**Keywords:** *Cardamine leucantha*, clonal plants, forest-floor herbaceous plants, genet assignment, population structure, RAD-seq, stoloniferous rhizome

## Abstract

To study the genetic structure of clonal plant populations, genotyping and genet detection using genetic markers are necessary to assign ramets to corresponding genets. Assignment is difficult as it involves setting a robust threshold of genetic distance for genet distinction as neighbouring genets in a plant population are often genetically related. Here, we used restriction site-associated DNA sequencing (RAD-seq) for a rhizomatous clonal herb, *Cardamine leucantha* [Brassicaceae] to accurately determine genet structure in a natural population. We determined a draft genome sequence of this species for the first time, which resulted in 66 617 scaffolds with N_50_ = 6086 bp and an estimated genome size of approximately 253 Mbp. Using genetic distances based on the RAD-seq analysis, we successfully distinguished ramets that belonged to distinct genets even from a half-sib family. We applied these methods to 372 samples of *C. leucantha* collected at 1-m interval grids within a 20 × 20 m plot in a natural population in Hokkaido, Japan. From these samples, we identified 61 genets with high inequality in terms of genet size and patchy distribution. Spatial autocorrelation analyses indicated significant aggregation within 7 and 4 m at ramet and genet levels, respectively. An analysis of parallel DNA microsatellite loci (simple sequence repeats) suggested that RAD-seq can provide data that allows robust genet assignment. It remains unclear whether the large genets identified here became dominant stochastically or deterministically. Precise identification of genets will assist further study and characterization of dominant genets.

## Teaser

Clonal plants produce genetically identical offspring through vegetative growth. A ‘ramet’ refers to a single physiological individual produced by clonal propagation; a ‘genet’ refers to a group of ramets that originate from a single seed. Here we investigated how ramets that belong to different genets distribute in a natural population of *Carrdamine leucantha* using a genome-wide SNP markers. We applied the method to 372 samples collected at 1-m interval grids within a 20 × 20 m plot, and identified 61 genets with high inequality in terms of genet size and patchy distribution.

## Introduction

Clonal plants produce genetically identical offspring through vegetative growth (van Groenendael and de Kroon 1990; Winkler and Fischer 1999). A ‘ramet’ refers to a single physiological individual produced by clonal propagation; a ‘genet’ refers to a group of ramets that originate from a single seed (Harper 1977; Jackson *et al.* 1986; Tuomi and Vuorisalo 1989). Some clonal plant species produce new ramets on the tips of stoloniferous rhizomes (Bell 1991; Klimeš *et al.* 1997); it is difficult to determine the distribution of ramets that belong to a particular genet without excavation (Silander 1979). Furthermore, ramets from multiple genets can be intermingled in some herbaceous species with elongated rhizomes; underground connections between ramets often decay within a few years (Eriksson and Jerling 1990; Kelly 1995; Araki and Ohara 2008). Therefore, genotyping and genet detection using genetic markers are necessary to assign ramets to corresponding genets (Widén *et al.* 1994; Arnaud-Haond *et al.* 2007). In clonal plants with rhizomes, there is a spectrum between the two extreme types. At one extreme are plants with long-lived short rhizomes, e.g. *Anemone nemorosa* (Stehlik and Holderegger 2000), *Maianthemum bifolium* (Honnay *et al.* 2006) and *Mercurialis perennis* (Vandepitte *et al.* 2009). At the other extreme are plants with short-lived long rhizomes. Particularly, in a type of life-cycle known as pseudo-annual (Whigham 1974; Kawano 1975, 1985), each ramet grows only for a single growing season, and mother ramets wither before daughter ramets emerge in the next season, e.g. *Uvularia perfoliata* (Kudoh *et al.* 1999).

*Cardamine leucantha* (Tausch) O. E. Schulz [Brassicaceae] is a pseudo-annual characterized by exceptionally long stoloniferous rhizomes. The length averaged 45.1 ± 21.5 (SD) cm in a natural population (Ida and Kudo 2009) and can reach 1.2 m in a transplant condition (Michiaki Tsujimoto, unpubl. data). The rhizomes between mother and daughter ramets become disconnected within 1–2 years. Therefore, a single genet of *C. leucantha* can develop into a group of multiple disconnected ramets, spreading tens of square metres or wider as shown in our previous study (Araki *et al.* 2017) using DNA microsatellite loci (simple sequence repeats, SSR) markers developed for *C. leucantha* (Araki *et al.* 2011). Contrastingly, *C. leucantha* seeds do not have particular measures for seed dispersion; most seeds are likely to be dispersed near the maternal plants. Therefore, probability of genet recruitment from seeds relative to that from clonal propagation should determine spatial clonal structures of populations. We expected a more intermingled structure comprised from closely related genets under higher relative recruitment from seeds. Contrarily, we expect low overlap levels between genets when establishment of new genets form seeds is rare. To test these expectations, we require a sensitive genetic marker that allows distinction between genets derived from seeds of a single plant.

The spatial genetic structure of clonal plant populations has been studied in a number of plant taxa, and clonal plant populations have been found to generally contain multiple genets with diverse genet distribution patterns (Parks and Werth 1993; Kudoh *et al.* 1999; Verburg *et al.* 2000; Araki *et al.* 2007; de Witte *et al.* 2012; Binks *et al.* 2015). Accordingly, genetic markers and analytical methods have been developed for more precise genet assignment (Kamvar *et al.* 2015; Bailleul *et al.* 2016). The analyses generally require distinction of closely related genotypes that co-exist within a relatively small study area (Meirmans and van Tienderen 2004; Kamvar *et al.* 2015; Bailleul *et al.* 2016). Two types of errors occur in genet assignment in such situations: the first is the assignment of ramets from different genets to the same genets; the second is the assignment of ramets from a single genet to distinct genets (Meirmans and van Tienderen 2004; Arnaud-Haond *et al.* 2007). The former arises when a polymorphism is shared among multiple loci between closely related genets. The latter error arises from the detection of somatic mutation, misidentification of heterozygous loci as homozygous for either allele, and genotyping error. Recently, whole genome sequencing with next-generation sequencers has become the most powerful method for genotyping individuals (Luikart *et al.* 2003); methods that utilize total polymorphism subsets have been developed to process large numbers of samples at lower cost. Restriction site-associated DNA sequencing (RAD-seq) is one such method that uses next-generation sequencing to obtain genome-wide single nucleotide polymorphisms (SNPs) from hundreds of individuals (Davey *et al.* 2010; Davey *et al.* 2011). It has been applied to many non-clonal species as a high-resolution marker for genotyping (Davey *et al.* 2011; Peterson *et al.* 2012); however, only a few studies have applied RAD-seq to clonal plants to estimate clonal structure within populations (Baughman *et al.* 2017; Ning *et al.* 2018).

Here, we applied RAD-seq to *C. leucantha*, aiming to accurately determine genet structure in its natural habitat of cool temperate deciduous forest. This species has several characteristics that make it well suited for the new method. The species is diploid (2n = 16, Al-Shehbaz *et al.* 2006a) and is expected to have a small genome (preliminary estimation, 270 Mbp). The species belongs to Brassicaceae, which contains a model species (*Arabidopsis thaliana*) used in studies of plant molecular genetics; this permits the use of a number of experimental protocols that have been developed for the species (Araki *et al*. 2011, 2017). Here, we determined a draft genome of *C. leucantha* onto which we mapped RAD-seq reads for precise SNP detection. We evaluated the genet assignment procedure by RAD-seq using a set of materials with known relationships between samples. We then applied RAD-seq and SSR analysis to a natural population of *C. leucantha*. RAD-seq provided robust genet assignment compared to SSR, and we evaluated detailed genet structure of the *C. leucantha* population.

## Methods

### Study species and sites

*Cardamine leucantha* is a herbaceous plant that grows on the floor and margins of deciduous forests of East Asia, including those in Japan, Korea, Mongolia, China and far-east Russia (Al-Shehbaz *et al*. 2006*a*, *b*; Song and Park 2011). A single flowering ramet elongates one upright stem (bolting) and produces an inflorescence of white, insect-pollinated flowers in spring. The stoloniferous rhizomes continue to elongate until the end of summer. Rhizomes rarely branch, and each rhizome produces a daughter ramet at the tip. One ramet may produce one or multiple rhizomes simultaneously.

**Figure 1.**
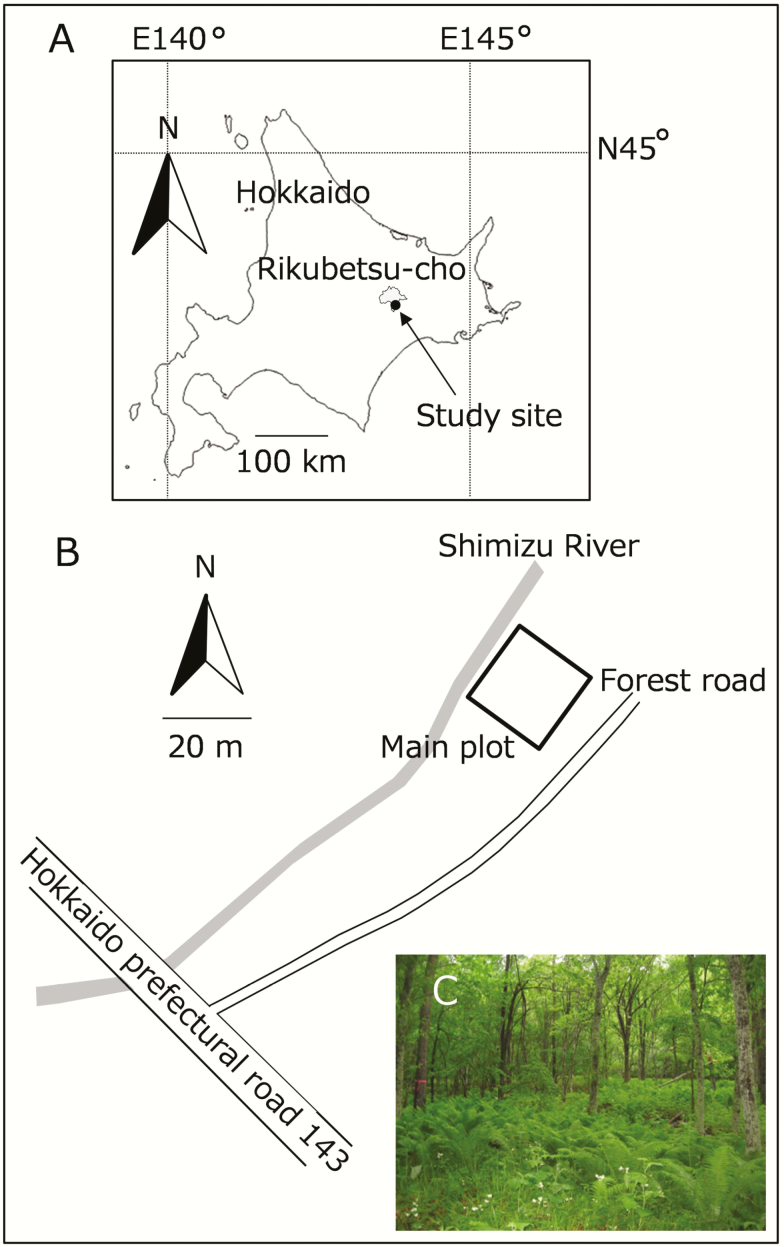
Map showing the location of the *Cardamine leucantha* study site at Rikubetsu-cho, Hokkaido, Japan (A), map showing the position of the 20 × 20 m main plot in the site (B) and a photograph of the study plot (C). In (A) and (B), the direction and distance are represented by upward arrows and bars, respectively.

The study was conducted in a cool-temperate deciduous forest along the Shimizu River in Rikubetsu, Hokkaido, Japan (N43°27′, E143°46′, alt. ca. 250 m, Fig. 1). At this site, a continuous population of *C. leucantha* extends over 3 ha along the stream. In the *C. leucantha* population, we primarily investigated a 20 × 20 m plot (referred to as the main plot, hereafter), which was established in 2010 in the previous study (Araki *et al.* 2017). The main plot was divided by grid lines at 1-m intervals, resulting in 441 crossing points in the plot (grid points hereafter). Ramets of *C. leucantha* formed a cover of varying density over the main plot. The number of ramets within 400 quadrats, which were divided by grid lines, ranged from 0 to 64, and the total number of ramets within the main plot was 6510 in 2012. Above- and below-ground temperatures ranged from –29.9 °C to 30.6 °C (4.8 °C average) and from –0.4 °C to 25.1 °C (8.0 °C on average), respectively, from 30 May 2012 to 14 June 2013 (Supporting Information—Method S1).

### Sampling to determine genet distribution within the main plot

We collected DNA samples to determine genet distribution within the main plot on 31 May 2012. The nearest flowering ramet within 0.5 m of each grid point was chosen. We selected a non-flowering ramet only when a flowering ramet was absent. For each selected ramet, a fresh leaf was harvested from the upper-most position along the stem. When the top leaf was small or damaged, the second leaf from the top was collected. The procedure is detailed in Supporting Information—Method S2.

### Sampling to determine the fine-scale spatial distribution of genets

To determine the fine-scale genet distribution, we collected DNA samples (on 13 June 2013). Based on the genet distribution determined for the main plot, six 1 × 1 m quadrats (Q1–Q6) were set in the main plot. Q1–Q4 and Q5–Q6 were set in the area occupied by two predominant genets, G1 and G3, respectively (genet IDs were determined the previous year). Leaves were sampled from each ramet within each quadrat and preserved as described for samples of the main plot. During sampling, the air temperature ranged from 15.3 to 20.7 °C. We conducted RAD-seq by adding the samples of genets G1–G3 from the previous analysis and then determined whether the ramets within the six quadrats belonged to any of the three genets.

### Preparation of materials to validate the genet assignment procedure

To validate the genet assignment procedure, we prepared materials belonging to two genets in the natural populations and ramets derived from them clonally and sexually. The detailed procedure is presented in Supporting Information—Method S3.

### Genome sequencing of *Cardamine leucantha*

For the mapping reference used in RAD-seq, the draft genome of *C. leucantha* was sequenced from a ramet belonging to the most predominant genet (Genet 1, G1). Silica-gel-dried leaf tissue was obtained from a potted plant grown in the garden of Center for Ecological Research (CER), Kyoto University, for 2 months after transplantation. The sample was then transferred to Zurich University for draft genome sequencing. Approximately 200 mg dried tissue was ground with glass beads (2–3 mm in diameter) using a tissue disruptor Silamat S6 (Ivoclar Vivadent, Schaan, Liechtenstein). DNA was extracted with a DNeasy Plant Mini Kit (Qiagen, Hilden, Germany) according to manufacturer’s instructions. The DNA concentration was measured with a Qubit dsDNA HS Assay (Invitrogen, Carlsbad, CA, USA). A TruSeq DNA fragment kit (Illumina, San Diego, CA, USA) was used for library synthesis, with an insertion size of 200 and 500 bp (50 bp +/- target fragment length). The DNA samples underwent Illumina HiSeq sequencing at the Functional Genomics Center (Zurich); the paired-end protocol had a read length of 101 bp.

We obtained 151 624 738 and 94 965 510 reads for libraries with 200- and 500-bp insertion size, respectively. We assembled reads using an ALLPATHS-LG assembler. Remapping the reads on the assembled genome resulted in mapping rates of 87.1 % and 91.6 % (132 021 494 and 94 965 510 reads) for libraries with insertion sizes of 200 and 500 bp, respectively [Bowtie 2, v2.2.3, default settings (Langmead *et al.* 2009)]. Genes were predicted using the gene prediction software AUGUSTUS (Stanke *et al.* 2004), and gene annotations for *A. thaliana* were obtained via one-way hit (OWH) and reciprocal best hit (RBH). OWH identified normal blast hits [query, predicted gene coding sequences (CDS) of *C. leucantha*; database, *A. thaliana* CDS (27 416 genes); E-value, 10^–15^]. RBH identified mutual best hit pair in two-way blast between *C. leucantha* and *A. thaliana* CDS. We calculated sequence homology as the mean percent identity between RBH-pairs. We also counted the number of highly aligned genes, where more than 90 % of the gene length was reciprocal between the corresponding CDS of *C. leucantha* and *A. thaliana*.

The *C. leucantha* raw genome sequence data was deposited in the DDBJ BioProject database (BioProject Accession number: PRJDB8595, BioSample Accession number: SAMD00180684).

### RAD-seq analysis

Genomic DNA was extracted from samples using the cetyltrimethylammonium bromide (CTAB) method adjusted for *C. leucantha* (Araki *et al.* 2011, modified from Doyle and Doyle 1987). The detailed procedure is described in Supporting Information—Method S4. Extracted DNA was stored at –20 °C until analysis.

RAD-seq analyses were used to assign genets for the main plot samples, fine-scale samples from the field site and selected parent–offspring (clonal and sexual) samples for method validation. DNA samples were used to prepare libraries for RAD-seq analyses as described in the Supporting Information—Method S5.

### Genet assignment from RAD-seq data

From the RAD-seq data, genet assignment was conducted through multiple steps described below (summarized in Supporting Information—**Table S1**). Raw reads (FASTQ files) obtained from sequencing were pre-processed by removing adapter sequences and low-quality fragments with the use of the software, trimmomatic v0.32 (Bolger *et al.* 2014). Trimmed reads were mapped to the *C. leucantha* draft genome using Bowtie 2 v2.2.3. Reads producing a unique best alignment to the genome, with three mismatches or fewer, were retained. Reads were stacked and contigs were built for each sample using Stacks v1.19 (Catchen *et al.* 2011). Contigs with a single diallelic SNP were used in the following analyses. Contigs with two or more SNPs were excluded; those with SNPs with more than two alleles were also excluded. We defined these contigs as polymorphic loci. Polymorphic loci with a minor allele frequency > 0.01 and with enough depth (i.e. minimum depth ≥ 10) for >90 % of samples were used for genotyping. For genotyping using RAD-seq analysis, restriction fragment bias would arise and therefore read depths vary across contigs or samples. This is seen especially under limited sequencing depth (Davey *et al.* 2013). Therefore, to assess the effect of minimum depth, other criteria for minimum depth were used, i.e. depth ≥ 5, ≥ 15, ≥ 20 and ≥ 30.

Based on similarity in the multi-locus genotypes (MLG) of ramets, they are assigned to a single genet when the similarity is higher than a certain threshold (genet-assignment threshold). However, somatic mutations and genotyping errors may cause MLG variation characterized by a small change in the number of loci (Meirmans and van Tienderen 2004; Arnaud-Haond *et al.* 2007). Therefore, to distinguish between MLG variation between and within genets, we determined a genet-assignment threshold using the following three procedures.

The first procedure involves the use of GENODIVE 2.0b17 (Meirmans and van Tienderen 2004) and the threshold determination method described below (referred to as GENODIVE method, hereafter). Based on a frequency distribution of pairwise genetic distance calculated using GENODIVE, we assumed that the first peak of small distance was derived from somatic mutations or genotyping errors within genets, and the second peak of long distance represented variation between genets (Meirmans and van Tienderen 2004). We selected a distance window between the two peaks where the assigned genet number was kept constant with increments of genetic distance and, then, set the genet-assignment threshold at the maximum distance in the window. Genets were then assigned based on the designated threshold using GENODIVE. The results presented in the main text are those analysed using the first method.

We also applied two R packages, implemented in R v3.4.1 (R Core Team 2017), RClone (Bailleul *et al.* 2016) and *poppr* (Kamvar *et al*. 2014, 2015, version 2.8.1 released in 2018) to assign clonal membership. RClone, simulates outcrossing and inbreeding events to select a threshold, but does not support missing data; however, in our dataset, missing data were negligibly low (less than 0.2 % for all samples). We used the *genet_dist ( )* function to compute pairwise genetic distances between ramets and the *genet_dist_sim ( )* function to compute genetic distances after a sexual reproduction event (both outcrossing and inbreeding) between ramets. The threshold was defined by the lowest distance among sexually produced seeds. The R package *poppr* is used to identify the largest gap between all putative thresholds. For this purpose, the *cutoff_predictor ( )* function was used to predict thresholds with *mlg.fliter ( )* function, which assigns genets based on genetic distances. We used the *diss_dist ( )* function to compute pairwise genetic distances between ramets based on relative dissimilarity reflecting the number of allelic differences between two individuals. When genets were assigned, we selected a conservative algorithm (farthest neighbour) for clustering.

To visualize the relationships between the different genets within the main plot, minimum spanning networks (MSN) were drawn using the *plot_poppr_msn ( )* function in the *poppr* package. We adopted genets which were identified by *poppr* for the analysis. Genetic distances between ramets were calculated by the *diss_dist ( )* function in the *poppr* package using SNPs with a depth of 10 and greater.

### Spatial autocorrelation, clonal diversity and genet size

Spatial autocorrelation was analysed based on whether a particular set of ramets belonged to the same genet. Moran’s *I* was calculated for each distance class at 1-m intervals, ranging from 0 to 18 m, for which we obtained a sufficient number of ramet pairs (*n* ≥ 2591) per distance class. Significance (*P < *0.05) was tested against a distribution of Moran’s *I* obtained by randomly permuting ramet positions 1000 times.

Clonal diversity was estimated by two parameters, namely *G*/*N* and Simpson’s index *D*. *G* represents the number of genotypes detected and *N* is the number of samples. *G*/*N* is a biased but frequently used estimator influenced by the number of ramets *N*. *D* is calculated as *D = 1* –Σqg^2^; where, qg is the frequency of the g-th genet. This index represents the probability that two ramets selected at random from a patch of *N* ramets are from different genets. Genet size inequalities were calculated using Gini coefficients (Weiner and Solbrig 1984).

### Spatial dependency of genetic distance and genetic variation within a single genet

Spatial autocorrelation was analysed based on the genetic distances between ramets belonging to the largest genet (G1). Moran’s *I* was calculated for each distance class at 1-m intervals, ranging from 0 to 18 m, for which we obtained sufficient numbers of ramet pairs (*n* ≥ 156) per distance class. The significance (*P < *0.05) was tested against a distribution of Moran’s *I* obtained by randomly permuting ramet positions 1000 times. We also calculated Moran’s *I* for genet G1 using SSR data described below.

To compare the variation within genets determined by RAD-seq and SSR analyses, for the top eight predominant genets, we calculated the number of polymorphic loci, the average number of genotypes per locus and the average number of alleles per locus.

### Spatial dependency of genet distribution

Spatial autocorrelation was analysed based on the genetic distances between genets. For genets with multiple ramets, a single ramet was selected for each genet, which was the nearest to the average coordinates of all ramets from the genet. Moran’s *I* was calculated for each distance class at 1-m intervals ranging from 0 to 18 m, for which we obtained sufficient numbers of ramet pairs (*n* ≥ 35) per distance class. The significance (*P < *0.05) was tested against a distribution of Moran’s *I* obtained by randomly permuting ramet positions 1000 times.

### SSR analysis

To evaluate the results of the RAD-seq analysis against the conventional analysis using DNA microsatellite loci, we performed a SSR analysis as previously described (Araki *et al.* 2011). For the main plot samples, 0.75–1.5 µL extracted DNA was amplified using 13 SSR markers. Multiplex polymerase chain reaction (PCR) was performed on each sample. Based on the annealing temperature and number of PCR cycles, five sets of multiplex primers [Cleu225 and Cleu244; Cleu267, Cleu642 and Cleu 648; Cleu387 and Cleu666; Cleu263, Cleu268, Cleu270 and Cleu452; and Cleu594 and Ctri174 (all primers with Cleu are described in Araki *et al.* 2011, and Ctri174 is firstly used here, F: 5´-TCCACTTGGAACCTTCATCT-3´, R: 5´-ACACACACACACTCTCTCTCTC-3´) were used to amplify 2–4 loci/set simultaneously. For size separation, 0.6–1.5 µL PCR product was electrophoresed using capillary electrophoresis on an ABI PRISM 3130 genetic analyser (Applied Biosystems, Foster City, CA, USA). Banding patterns were scored and genotyping was performed in a semi-automated manner using GeneMapper 3.7 (Applied Biosystems). Genet assignment was conducted with the same methods used for RAD-seq analysis, i.e. GENODIVE, RClone and *poppr* methods. In the GENODIVE method, pairwise genetic distances were calculated using a stepwise allele model in the SSR analysis instead of an infinite allele model in the RAD-seq analysis (Meirmans and van Tienderen 2004).

## Results

### Genome sequencing

Genomic DNA from one *C. leucantha* individual (Genet 1, G1) was sequenced to obtain reference sequences for RAD-seq analyses. We assembled a total of 307 756 952 reads, resulting in 66 617 scaffolds with 188 557 bp max length and N_50_ = 6086 bp. Estimated genome size was 253 130 468 bp, which was close to our genome size estimation (ca. 270 Mbp) based on flow cytometry. The heterozygosity rate was 1.11 %. The number of predicted genes was 79 324; 75 434 genes had a CDS longer than 200 bp. Gene annotation against the *A. thaliana* database revealed 31 198 and 19 203 OWH and RBH genes, respectively, and 9750 highly aligned genes. For RBH pairs, sequence homology was 89.83 % on average. These data suggest that the assembly is adequate for RAD-seq analysis, for which the mapping of short-reads from different individuals is required.

### Validation of the genet assignment procedure

**Figure 2.**
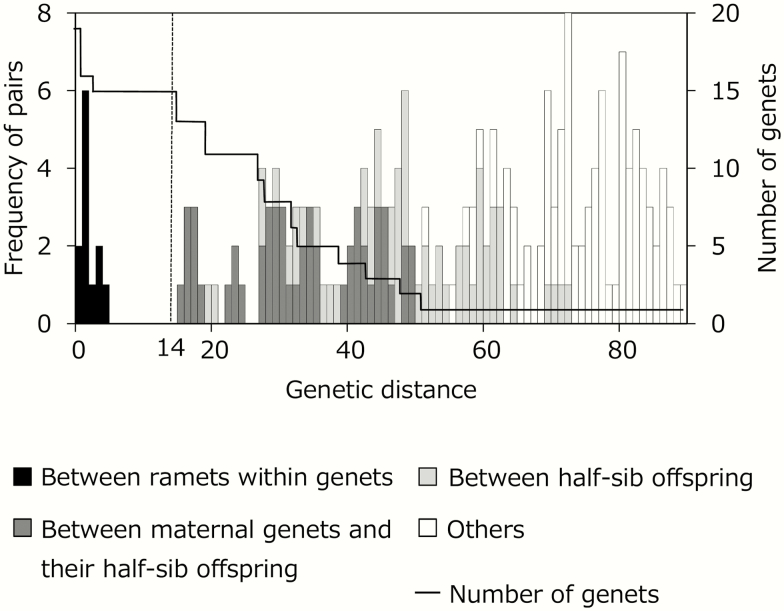
Validation of the genet assignment procedure applied for the restriction site-associated DNA sequencing (RAD-seq) analysis in this study. Frequency distributions of pairwise genetic distances between ramets with known genetic relationships are shown by bars with different shades. Pairs between clonal ramets within genets, between maternal ramet and their half-sib offspring, between half-sib offspring and between un-related genets are represented in black, dark-grey, grey and open bars, respectively. A solid line represents the number of genets when the corresponding genetic distances were used as a threshold for assignment. A dashed line represents the selected threshold of genetic distance. Genetic distances were calculated using 264 single nucleotide polymorphisms (SNPs) with a depth of ≥ 10 and based on an infinite allele model.

Firstly, we confirmed that our genet assignment procedure (described in the Materials and Methods section) was able to distinguish ramets that belong to different genets even when they were closely related and to assign clonal ramets to a single genet. Pairwise genetic distances between ramets were calculated using 264 contigs (SNPs), which were polymorphic loci, single diallelic, with read depths ≥10. Pairwise genetic distance ranged from 0 to 89 and presented a bimodal frequency distribution (Fig. 2). Genetic distances between clones within genets (0–4) were distinctive from those between genets (15–89, Fig. 2). The genet assignment threshold (=14), determined by the procedure, successfully distinguished clones within the genets from related genets, even when they were half-sibs (Fig. 2). When we applied the threshold, the number of assigned genets for ramet samples was 15, which corresponded to the known number of genets. The calculated genetic distances increased with decreasing relatedness, i.e. the values were 15–45, 19–72 and 51–89 between maternal clones and their half-sib offspring, among half-sib offspring and between G1 and G3 derived plants, respectively (Fig. 2).

### Genet assignment in the natural population

**Figure 3.**
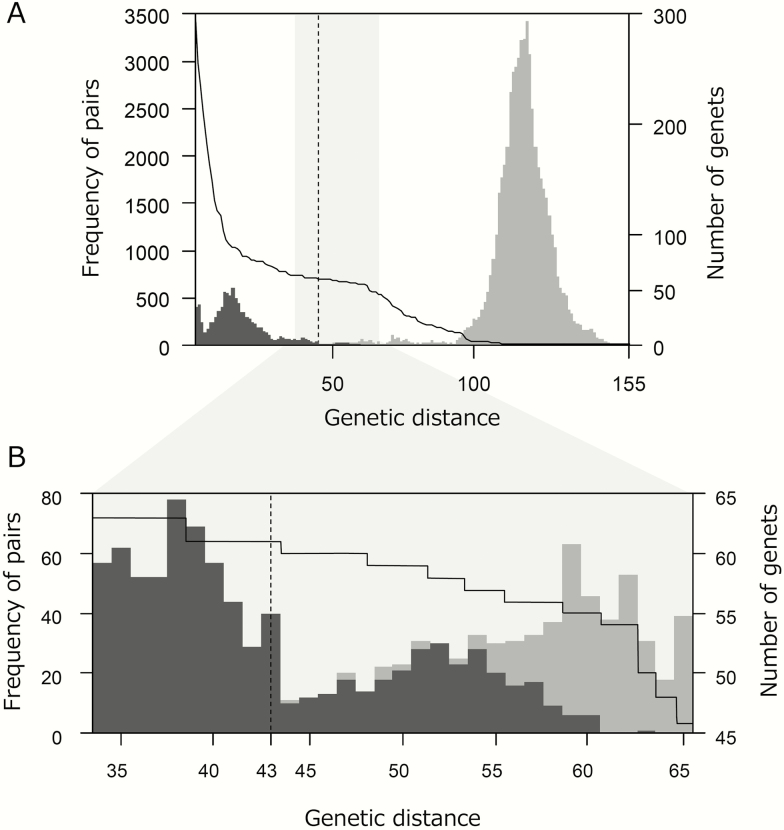
Genet assignment of the study plot in a natural population of *Cardamine leucantha.* Frequency distribution of pairwise genetic distances between ramets calculated from SNPs in the RAD-seq analysis (grey bars) and number of genets when the corresponding genetic distances were used as a threshold for genet assignment (solid line). A ramet with a genetic distance smaller than the threshold of at least one of the ramets within a certain genet was assigned to the genet. A dashed line represents the threshold of genetic distance applied for genet assignment (= 43). Frequency distribution of all pairs (A) and an enlarged view near the applied threshold (B, corresponding to the right grey area in A) are shown. Ramet pairs represented by dark grey bars were assigned to members of the same genet. Ramet pairs represented by grey bars were considered to be from different genets. Genetic distances were calculated using 363 SNPs with a depth ≥ 10 based on an infinite allele model. For this analysis, 384 selected samples obtained at the grid points of the study plot were used, except for those with less than 90 % of selected SNPs (12 samples).

RAD-seq analyses of the main plot obtained data on 372 of 394 sampled ramets (for 17 of 22 excluded ramets, we assigned their genet identity in a separate analysis). The stacks read number per analysed ramet ranged from 53 640 to 707 514. From the stacks reads from initial samples, 179 265 contigs in total were mapped on to the *C. leucantha* genome. The 363 contigs (SNPs) with a depth ≥10 were identified as polymorphic loci applicable for further analyses (defined in the Materials and Methods). Pairwise genetic distance between ramets ranged from 0 to 155 and followed a clear bimodal frequency distribution (Fig. 3). Applying the GENODIVE method, the genet-assignment threshold of genetic distance resulted in a distance 43; the number of assigned genets was estimated as 61 (Fig. 3 and Supporting Information—**Table S2**). In the analyses using RClone and *poppr*, we identified similar numbers (68 and 67, respectively) and similar patterns of spatial distribution of genets (Supporting Information—**Figure S1**).

### Effects of minimum depth on the filtering of SNP loci

To assess the effects of minimum depth on the filtering of SNP loci, we performed genet assignment using other criteria for minimum depth, i.e. depth ≥ 5, ≥ 15, ≥ 20 and ≥ 30 in addition to ≥ 10 (criteria used for all analyses). Overall, 621, 363, 217, 138 and 69 contigs (SNPs) were identified as polymorphic loci for further analyses (defined in the Materials and Methods) at depths of ≥ 5, ≥ 10, ≥ 15, ≥ 20 and ≥ 30, respectively (Supporting Information—**Table S2**). Pairwise genetic distance between ramets ranged from 0 to 247, 155, 93, 64 and 34 at depths of ≥ 5, ≥ 10, ≥ 15, ≥ 20 and ≥ 30, respectively (Supporting Information—**Table S2**). The genet-assignment threshold of genetic distance resulted in distances of 96, 43, 31, 16 and 5, and the numbers of assigned genets were estimated as 56, 61, 58, 58 and 59, at depths ≥ 5, ≥ 10, ≥ 15, ≥ 20 and ≥ 30, respectively (see Supporting Information—**Table S2**). The difference in minimum depth had little effect on the number of assigned genets, clonal diversity index (*G*/*N* and Simpson’s *D*) and genet size inequalities (Gini coefficients). The *G*/*N* value was estimated to be 0.15 in the ≥ 5 depth analyses and 0.16 in the other depth analyses (Supporting Information—**Table S2**). Simpson’s *D* value was estimated to be 0.86 for a depth ≥ 10 and 0.85 for other depth criteria (Supporting Information—**Table S2**). The Gini coefficient was estimated to be 0.71 at a depth ≥ 5 and 0.72 at the other depth criteria (Supporting Information—**Table S2**).

### Genet distribution and clonal structure within the main plot

**Figure 4.**
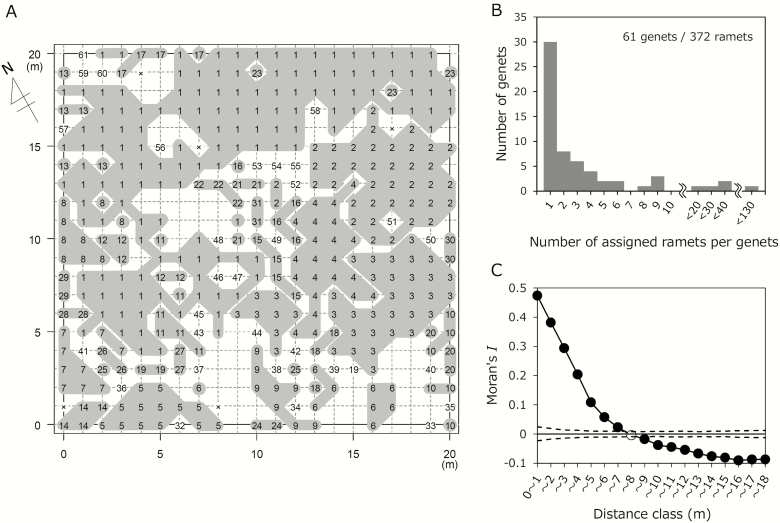
Genet structure of the main plot determined by the RAD-seq analysis (A–C). Spatial distribution of genets (A), frequency distribution of ramet number per genet (B) and spatial aggregation of ramets belonging to the same genet shown by the spatial autocorrelation analysis on the ramet distribution (C) are listed. In (A), ramets were sampled at grid points of the main plot (crossing points of grid lines set at 1 m intervals) in 2012. Different numbers represent different genets. Genets were ranked by the number of assigned ramets in the samples. Genets consisting of at least two ramets are represented by grey shades. Ungenotyped samples are shown by ‘x’. The orientation of the plot is shown by an upward arrow. In (C), spatial autocorrelation was analysed based on whether a particular set of ramets belonged to the same genet. Solid lines and circles represent Moran’s *I* at different distances. Dotted lines represent 95 % confidence limits in the null model based on 1000 permutation tests. The filled circles represent significant (*P < *0.05) deviations from the null model.

RAD-seq analyses identified 30 genets with a single ramet sample for each, and 31 genets with multiple ramets that showed patchy distribution (Fig. 4A and B). Especially, ramets of the most predominant genet (G1) formed a wide patch, occupying 34 % of the grid points. Spatial autocorrelation calculated by the genet identity of all ramets declined with increasing distance and was positively significant (*P <* 0.05) up to 7 m (Fig. 4C). Spatial autocorrelation calculated using genetic distance for representative ramets of all genets declined with increasing distance, was positively significant up to 4 m and was negatively significant for the 11–12 and 12–13-m distance classes (*P < *0.05, Supporting Information—**Figure S2A** and **B**). The minimum spanning network (MSN) generated by MSN function in *poppr* indicated that predominant genets were not always related to each other and were spread all over the MSN (Supporting Information—**Figure S3**). Some sets of predominant genets—G2, G4 and G6 and G5, G7 and G9—located proximately from each other both on the MSN (Supporting Information—**Figure S3**) and the genet distribution map (Fig. 4). Many of the small genets were closely related to the neighbouring predominant genets (Supporting Information—**Figure S3**).

**Figure 5.**
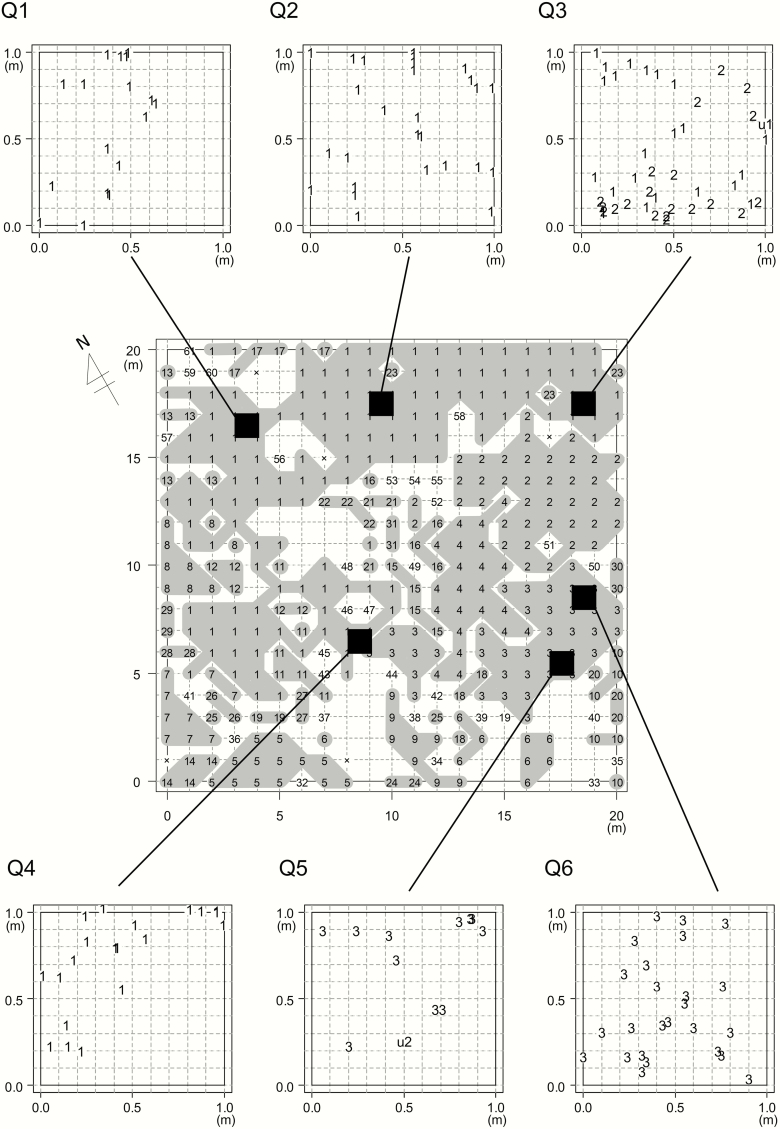
Fine-scale spatial distribution of genets within six 1 × 1 m quadrats (Q1–Q6). Different numbers represent different genets. The locations of six quadrats are shown on the spatial distribution of genets determined in the previous year. Q1–Q4 and Q5–Q6 were set in areas where genets G1 and G3 were dominant, respectively. Q3 was set near the border with genet G2. We performed RAD-seq by adding the samples of genets G1–G3 from the previous analysis, and then determined whether the ramets within the six quadrats belonged to any of the three genets. As a result, two genets were detected (u1 and u2), which were not assigned to G1–G3. We used 472 SNPs with a depth of 10.

We used 472 SNPs to analyse the fine-scale spatial distribution within six 1-m^2^ quadrats. For Q1–Q4, located in the area dominated by G1, three contained only G1 ramets (Fig. 5). At Q3, we identified G2 ramets spatially intermingled with G1 ramets (Fig. 5). For Q5 and Q6, located in the area dominated by G3, both quadrats consisted of G3 ramets. Two unique genets that were not assigned to G1–G3 were detected in Q3 and Q5, respectively (Fig. 5).

In SSR analyses, the number of alleles per locus ranged from 2 to 12 (5 on average) in 13 SSR loci, and 348 ramet samples were genotyped successfully. Pairwise genetic distance between ramets ranged from 0 to 61 (Supporting Information—**Figure S4A**). Although frequency distributions of pairwise genetic distance presented bimodal patterns, the gap between the first and second peaks was not clear compared with that of RAD-seq analyses (Supporting Information—**Figure S4A**). Therefore, the genet-assignment threshold of genetic distance was also indeterminate, at around three to four by the GENODIVE method (Supporting Information—**Figure S4A**). We identified 110 and 94 genets when we set genetic distances 3 and 4 as the genet-assignment threshold, respectively (Supporting Information—**Figure S4B**), which was greater than the ramet number estimated by the RAD-seq analyses. More genets were identified with the assignments by RClone and *poppr* (125 and 127, respectively, Supporting Information—**Figure S4E** and **F**). The size distribution and locations of genet patches identified by SSR analyses were similar to those identified by the RAD-seq analysis (Supporting Information—**Figure S4B–F**).

### Spatial dependency and genetic variation within a single genet

Pairwise genetic distance between 128 ramets within genet G1 ranged from 0 to 63, with unimodal frequency distribution (see Supporting Information—**Figure S5A**). No significant correlation was detected between genetic and spatial distances (Supporting Information—**Figure S5B**). No spatial autocorrelation was detected within the genet G1 patch (Supporting Information—**Figure S5C**). Similar results were obtained for the second and third predominant genets, G2 and G3 (data not shown). In the same analysis for 128 ramets within genet G1 using SSR data, spatial autocorrelation was positively significant (but weak) up to 4 m for all 13 loci and up to 1 m for the locus Cleu270 and Cleu648 (Supporting Information—**Figure S6**).

In the comparison of genetic variation within the top eight largest genets in the results of the RAD-seq and SSR analysis, the value of the average number of genotypes per locus and the average number of alleles per locus was larger in SSR (1.31–3.00 and 1.62–2.77, respectively) than that in RAD-seq (1.02–1.35 and 1.18–1.31, respectively; Supporting Information—**Table S3**). The value of three parameters of the most predominant genet G1 was larger compared to other genets (Supporting Information—**Table S3**).

## Discussion

The spatial genetic structure of forest floor plants is determined by genet and ramet demography, which is affected by the micro-environment dynamics on the forest floor (Eriksson and Bremer 1993; Kudoh *et al.* 1999; Vandepitte *et al.* 2009). High clonal diversity was detected in the study population of *C. leucantha*; 61 genets were identified in the 20 × 20 m study plot, even for selected samples at 1-m intervals (372 of 6510 total ramets within the plot). This indicates that sexual reproduction through seeds and successful recruitment of new genets occur. Analyses of all ramets within the six 1 × 1 m quadrats revealed a few unique genets mixed within occupying predominant genets. This also indicates the recruitment of new genets from seeds, although the rate of successful establishment per seed may be low. We observed pollinators, such as bumblebees, syrphus fly and white butterflies, frequently visiting flowers of *C. leucantha* in the study plot; the average fruit set of flowering ramets was approximately 28 % (Tsujimoto *et al.*, unpubl. data). More seedlings were found at the edge of the forest or along the forest road than within the main plot. In *C. leucantha*, higher fruit sets (approximately 50 %) are reported in the light gap sites compared with under the canopy (approximately 17 %) in the other population (Ida and Kudo 2009). Our data, however, suggest that new genets are recruited at a slower rate, even in the dense population of *C. leucantha*. The recruitment of new genets from seeds has also been reported in a dense clonal patch of forest floor plants, *Uvularia perfoliata* (Kudoh *et al.* 1999), *Anemone nemorosa* (Stehlik and Holderegger 2000), *Circaea lutetiana* (Verburg *et al.* 2000) and *Sasa kurilensis* (Matsuo *et al.* 2018).

Conversely, high inequality of genet size and the patchy distribution of genets indicate that clonal ramet production is another major determinant of genetic structure. For example, the largest genet (G1) occupied 34 % of the total sample and extended at least 20 m in distance. Some of the largest genets are likely to have persisted and propagated clonally within the population for several decades or longer. High clonal diversity and genet size inequalities have been reported in forest-floor plants, which reproduce clonally by elongating stolon or rhizomes (Kudoh *et al.* 1999; Araki *et al.* 2007; Wiberg *et al.* 2016).

Spatial autocorrelation analysis of genet patches revealed strong aggregations of genetically similar ramets within 7 m. The 7-m range may be due to the average size of the patch where a single genet can expand. Comparing genet patches, genet-level autocorrelation was detected over a small area (up to 4 m). Similar spatial patterns have been reported in *Lepidosperma* sp. (Binks *et al.* 2015) and *Halophila ovalis* (Xu *et al.* 2019). Our results suggested that small genets were genetically similar to neighbouring predominant genets, probably representing recruitment from seeds produced by the proximal large genets. The predominant outbreeding of the species may prevent the development of highly aggregated patches of closely related genets, but seed production by predominant genets is likely the major source of genet recruitments in the *C. leucantha* population. In one of a few studies on spatial genetic structure within a plant population using RAD-seq, a population of a wetland tussock species, *Blysmus sinocompressus*, was characterized by genet size inequalities and recruitment of seedlings that are offspring of a few original genets (Ning *et al.* 2018).

Although some variation in RAD-seq SNPs was found within predominant genets, we found no aggregations of ramets with a shared genotype within the largest genet, G1; therefore, we found no evidence for a structure derived from somatic mutations in RAD-Seq markers. Contrastingly, we found weak spatial structure in SSR locus; one possible explanation of this observation is a combination of high somatic mutation rate of the marker and aggregations of ramets by clonal growth. In *Prunus avium*, a patchy structure formed by ramets with a common somatic mutation has been reported (Jarni *et al.* 2015). Wang *et al.* (2019) also reported the distribution of somatic mutations within branches, roots, or runners using individuals of eight plant species.

The main plot in the present study was the same as that used to analyse genetic structure by eight SSR markers (Araki *et al.* 2017). Araki *et al.* (2017) collected DNA samples in 2010; we collected samples in 2012. We detected a similar pattern to that previously reported in terms of the size distribution and location of the large-sized genet. The number of assigned genets differed between the two studies; we identified 61 and 94–110 genets through RAD-seq and SSR analyses, respectively. Araki *et al.* (2017) identified 137 genets within the main plot. In our SSR analyses, the number of estimated genets varied depending on the assignment procedure; application of RClone and *poppr* increased the number of genets to values near those estimated by Araki *et al.* (2017). The number of assigned genets was more stable with RAD-seq depending on the method used; with, 61, 68 and 67 genets identified with the GENODIVE, RClone and *poppr* methods, respectively. This is mainly because the gap between the two frequency distribution peaks of pairwise genetic distances, i.e. the distinction of genetic distances within and between genets was relatively small when the SSR method was used, compared with RAD-seq. Furthermore, the RAD-seq analyses allowed us to evaluate genetic relatedness between genets; the small genets appeared to be derived from neighbouring predominant genets. In the validation of the genet assignment procedure, estimated genetic distances increased with decreasing relatedness. The RAD-seq analyses applied in the present study had high resolutions, which were able to distinguish genets from a single half-sib. Similar to our results, Thrasher *et al.* (2018) reported that relatedness value (*r*) calculated from a bird population (variegated fairy-wren, *Malurus lamberti*) increased with increasing actual relatedness using RAD-seq analysis.

For genotyping using RAD-seq analysis, the risk of restriction fragment bias under limited sequencing depth has been noted (Davey *et al.* 2013). Xu *et al.* (2014) estimated that at least three and four reads are required to determine genotypes, and these values are lower than 5–10 on average, in many studies within natural populations (Baughman *et al.* 2017; Wang *et al.* 2017) in balance with the number of available loci. In our study, at depths ≥ 5 and ≥ 30, the results were similar and robust in the assignment and analyses of genet distribution within the main plot. Furthermore, in the RAD-seq analysis, we aimed to accurately identify genets and strongly selected SNPs used for genet assignment. Finally, the number of SNPs used dropped to 0.2 % of the total (363, depth ≥ 10). This number is less than 2334, 7256 and 19 501 used for the genet assignment by RAD-seq of a moss and a sedge plant by Baughman *et al.* (2017) and Ning *et al.* (2018). Conversely, the number is higher than the 144 used for the same purpose from a species of *Sasa* by MIG-seq (another marker) in a study by Suyama *et al.* (2015).

In conclusion, by using RAD-seq we identified high inequality in genet size and patchy distribution of genets within a *C. leucantha* population. The resolution of the marker was proven to be distinguishable between a maternal plant and its half-sib offspring. The small genets are likely derived from seed production of neighbouring predominant genets. The results suggested that ramet production ultimately enhances successful offspring production through seed production by increasing genet size. Size variation is expected to derive from genet age (McLellan *et al.* 1997; de Witte *et al.* 2012, but see Tanner 2001; Ally *et al.* 2008), environmental heterogeneity (Harper 1977; de Steven 1989; Vandepitte *et al.* 2009) and other stochastic processes (McLellan *et al.* 1997; Verburg *et al.* 2000). Additionally properties of ramet production and growth rate—the number of ramets produced and the length of stolons or rhizomes—can be determined genetically, at least in part (de Kroon *et al.* 1994; Stuefer *et al.* 2009). Whether genetic variation of the ramet production trait can determine spatial genet structure is an open question; thus, precise identification of genets will aid further efforts to characterize dominant genets.

## Supporting Information

The following additional information is available in the online version of this article—

Method S1. Temperature measurement at the study site. 

Method S2. Leaf sampling from the main plot. 

Method S3. Preparation of materials to validate the genet assignment procedure. 

Method S4. DNA extraction. 

Method S5. Preparation of RAD-seq library.

**Figure S1**. Spatial distribution of genets within the main plot determined by RAD-seq using the method presented in the main text (GENODIVE) and two R packages, RClone and *poppr*. 

**Figure S2**. Spatial dependency of genet distribution based on genetic distances between genets.

**Figure S3**. Minimum spanning tree (MSN) of genets within the main plot drawn by an R package, *poppr*. 

**Figure S4**. Genet assignment of ramets and genet structure of the main plot determined by the SSR analysis.

**Figure S5**. Genetic distance between ramets in RAD-seq within the largest genet (G1) and its spatial dependency.

**Figure S6**. Spatial dependency between ramets in SSR loci within the largest genet (G1).

**Table S1**. Overview of the genet assignment procedure from the RAD-seq data.

**Table S2**. Effects of minimum depth in the filtering of SNP loci on the results of the RAD-seq analysis. 

**Table S3**. The genetic variation within the top eight largest genets in the RAD-seq and SSR analysis.

plz080_suppl_Supporting_Information

## Sources of Funding

This study was supported by Japan Society for the Promotion of Science (JSPS), Grant-in-Aid for Scientific Research (S) no. 26221106 and Japan Science and Technology Agency (JST), Core Research for Evolutional Science and Technology (CREST) no. JPMJCR15O1 to H.K., Swiss National Science Foundation and JST CREST no. JPMJCR16O3 to K.K.S., URPP Evolution in Action of University of Zurich to R.S.-I. and K.K.S. and Grant-in-Aid for Japan Society for the Promotion of Science Fellows 14J00722, MEXT to M.T.

## Contributions by the Authors

M.T., K.S.A. and H.K. designed the research. M.T. and K.S.A. conducted the field measurement and sampling. M.T., K.S.A. and M.N.H. conducted the laboratory experiment. M.T., K.S.A., A.J.N. and M.Y. conducted the data analysis. S.A., M.H., R.S.I., K.S.S. and J.S. conducted draft genome sequencing of *Cardamine leucantha*. M.T. and H.K. wrote the manuscript. H.K. supervised the study. All authors discussed the results and approved the manuscript.

## Conflict of Interest

None declared.
