# Supplementary material for: Genet assignment and population structure analysis in a clonal forest-floor herb, Cardamine leucantha, using RAD-seq
Source: AoB Plants. 2019 Dec 20;12(1):plz080. doi: 10.1093/aobpla/plz080 (PMC6983914; doi:10.1093/aobpla/plz080)
Supplement: plz080_suppl_Supporting_Information [file plz080_suppl_supporting_information.pdf]

Supporting Information

Genet assignment and population structure analysis in a clonal forest-floor herb, *Cardamine leucantha*, using RAD-seq

Michiaki Tsujimoto, Kiwako S. Araki, Mie N. Honjo, Masaki Yasugi, Atsushi J. Nagano, Satoru Akama, Masaomi Hatakeyama, Rie Shimizu-Inatsugi, Jun Sese, Kentaro K. Shimizu, and Hiroshi Kudoh

Supplementary Information Methods

Method S1. Temperature measurement at the study site

Method S2. Leaf sampling from the main plot

Method S3. Preparation of materials to validate the genet assignment procedure

Method S4. DNA extraction

Method S5. Preparation of RAD-seq library

Supporting Information Figures

Figure S1. Spatial distribution of genets within the main plot determined by RAD-seq using the method presented in the main text (GENODIVE) and two R packages, RClone and *poppr*.

Figure S2. Spatial dependency of genet distribution based on genetic distances between genets.

Figure S3. Minimum spanning tree (MSN) of genets within the main plot drawn by an R package, *poppr*.

Figure S4. Genet assignment of ramets and genet structure of the main plot determined by the SSR analysis.

Figure S5. Genetic distance between ramets in RAD-seq within the largest genet (G1) and its spatial dependency.

Figure S6. Spatial dependency between ramets in SSR loci within the largest genet (G1).

Supporting Information Tables

Table S1. Overview of the genet assignment procedure from the RAD-seq data.

Table S2. Effects of minimum depth in the filtering of SNP loci on the results of the RAD-seq analysis.

Table S3. The genetic variation within the top eight largest genets in the RAD-seq and SSR analysis.

Supporting Information Methods

Method S1. Temperature measurement at the study site.

Temperatures were measured by two temperature loggers (HOBO Water Temperature Pro v2, Onset Computer Co., Bourne, MA, USA) wrapped by aluminium foil. They were placed at the surface of a tree trunk at a height of 1.2 m and in the ground at a depth of 10 cm from the ground surface, to measure above- and below-ground temperatures, respectively.

Method S2. Leaf sampling from the main plot.

One or two leaflets were sampled depending on the leaf size. We did not sample at grid points that had no ramets within 0.5 m. Total number of sampled ramets was 394. Each sampled leaf was put in a thin paper bag (9.5 × 7 cm, product originally for tea extraction) immediately after sampling. Samples in the tea bags were placed in plastic bags with silica gel within five hours. The air temperature during the sampling was cool, ranging from 15.4ºC to 23.7ºC; all leaf samples were fresh when they were placed in silica gel. Dried samples were kept in the plastic bags with replaced fresh silica gel at an air-conditioned room (set at 25ºC) until DNA extraction.

Method S3. Preparation of materials for validation of the genet assignment procedure.

For validation of genet assignment procedure, we prepared materials for two genets and ramets derived from them clonally and sexually. We collected two ramets (each of the two belonged to either G1 or G3) from the main plot on Jun 13, 2012. We transplanted them to the garden in the Center for Ecological Research, Kyoto University (Otsu, Shiga Pref., Japan; N34°58′, E135°57′; alt. ca. 140 m). The multiple clonal ramets from the two original plants were obtained, and they produced seeds in the spring of 2013. On May 18, a fresh leaf at the top along the stem was harvested from four clonal ramets for each genet. On Jun 17, we collected seeds as maternal half-sibs of G1 or G3 ramets (potential pollen donors were ca. 50 genets transplanted into the same garden). Seeds were stored in the tea bags placed in plastic bags at a freezing room (set at 4 ºC) until seeding. On Sep 13, 2014, they were seeded on quartz sand in plastic petri dishes and

watered every two days. Petri dishes were placed in a growth chamber (Biotron LH300, NK Systems, Tokyo, Japan) with 12h/12h day/night cycle at 10ºC/20ºC day/night temperatures. Two and eleven seedlings from G1 and G3, respectively, were obtained, and these were transplanted into plastic pots with compost after 7-12 days after germination. The pots were placed in the above growth chamber with 12h/12h day/night cycle at 20ºC/20ºC day/night temperatures. Light was supplied by fluorescent lamps, and light intensity was ca. 50 $\mu Mm^{-2}s^{-1}$ at the pot surface level. On Nov 27, 2014, an uppermost fully expanded leaf was collected from each of 13 plants for DNA extraction.

Method S4. DNA extraction

Genomic DNA was extracted using the cetyltrimethyl ammonium bromide (CTAB) method adjusted for *C. leucantha* (Araki *et al*. 2011, modified from Doyle and Doyle 1987). Approximately 3-5 mg dried leaf per sample and 200 µl CTAB buffer was put into a plastic tube with a metal bead (Yasui Kikai Co., Osaka, Japan). Samples were pulverized by the Multi-beads Shocker (Yasui Kikai Co.). The extract mixture was incubated for 20 minutes at 50ºC. After incubation, the extract mixture was spun at 2,500 rpm for 10 minutes at 20ºC, and the supernatant was transferred to a new tube. For each sample, 200 µl chloroform: isoamyl alcohol (24:1) was added to the supernatant and mixed by decanting. After mixing, the samples were spun at 2,500 rpm for 10 minutes at 20ºC. The supernatant was transferred to a new tube and precipitated by adding 100 µl isopropanol. The mixture was spun at 2,500 rpm for 40 minutes at 4ºC. The precipitate (the extracted DNA) was washed twice with 100 µl 70% ethanol. After removing residual ethanol by vaporizing at an air-conditioned room (set at 25ºC), the extracted DNA was dissolved in 20 µL TE buffer [1 mM Tris-HCl, 0.1 mM ethylene-diamine-tetra-acetic acid (EDTA), pH 8]. Extracted DNA samples were stored at –20°C until analysis.

Method S5. Preparation of RAD-seq library

We applied RAD-seq analyses to assign genets for the main plot samples, the fine-scale samples from the field site, and the selected parent-offspring (clonal and sexual) samples for the method validation. The procedures were different in several points for

the former one and the latter two. We describe the procedure for the first set of samples and then explain the different points for the second and third sets of samples.

For samples of the main plot, 1 µL DNA per sample was used without diluting. DNA concentration of selected four samples from distant positions within the plots quantified by Qubit 2.0 Fluorometer using Qubit DNA Assay Kit (Thermo Fisher Scientific, Waltham, MA, USA) ranged from 30 to 50 ng /µl. Extracted DNA was digested with *Bgl*II and *Nde*I restriction enzymes that recognize 4- and 6-base elements, respectively. Adaptors specified to each digested side with in-complementary sequences were ligated. Samples were then amplified by primers (3′-CAAGCAGAAGACGGCATACGAGATXXXXXXGTGACTGGAGTTCAGACGTGT-5′, 3′-AGCACATCCCTTTCTCACATTAGAGCCACCAGCGGCATAGTAA-5′) that were complementary to one strand of each adaptor. Six-base pair sequence index tags (XXXXXX in the above primer sequence) were included in the primer of 3′ side to distinguish each sample. All amplified products were pooled into two sets (192 samples/set) and size-selected by gel electrophoresis using the E-Gel system (Invitrogen). DNA fragments were collected at every 50-bp size intervals from 250 to 450 bp. Their quantity and integrity were assessed by Qubit (Life Technologies) and High sensitivity DNA assay of Agilent 2100 Bioanalyzer (Agilent, Waldbronn, Germany). Based on the measurements, we selected samples containing 350 bp fragments (assessed with Agilent 2100 Bioanalyzer) as libraries; the concentration was more than 0.5 ng/µl (assessed with Quantus) for the both sets. Libraries were submitted for Illumina HiSeq 2000 sequencing at the Beijin Genomics Institute (BGI, Shenzhen, China). The sequencing consisted of two lanes (192 samples /lane) with a read length of 49 bp

For other samples (the fine-scale samples from the field site and the selected samples for validation of the genet assignment), DNA concentration was checked for all samples using QuantiFluor dsDNA system (Promega, Madison, WI, USA) and Tecan spectra fluor plus photometer (Tecan, Crailshein, Germany) with calculation software (X-Fluor, Tecan). Samples with DNA concentrations higher than 5 ng/µl (156 of 192 samples of the fine-scale samples from the field site) and 7 ng/µl (16 of 21 samples of the selected samples for the method validation) were diluted to 5 or 7 ng/µl by sterilized

water. The remaining samples were used without dilution. For these sets of samples, we chose size-selected samples that contained 300-bp fragments (assessed with Agilent 2100 Bioanalyzer) as libraries and whose concentrations were more than 1.0 ng/μl (assessed with Quantus). Libraries were submitted to Macrogen (Seoul, Korea) for Illumina HiSeq 2000 sequencing, with one lane (192 samples/lane) against the fine-scale samples from the field site and with 0.1 lane (192 samples/lane) against the selected samples for the method validation with a read length of 51 bp.

Supporting Information Figures

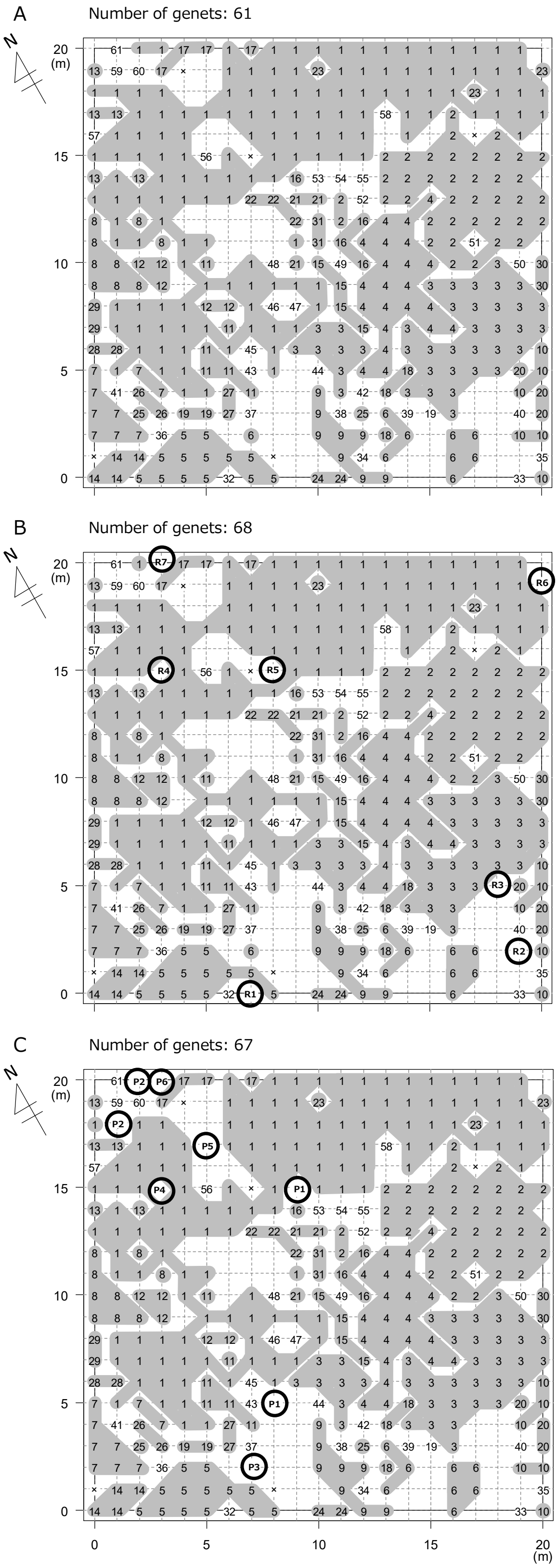

Figure S1 Spatial distribution of genets within the main plot determined by RAD-seq using the method presented in the main text (GENODIVE, A) and two R packages, RClone (B) and *poppr* (C). The total number of assigned genets are shown at the top right of each figure. Different numbers represent different genets. Genets were ranked by the number of assigned ramets in the samples based on the results from GENODIVE. In (B) and (C), ramets which assigned differently compared with the results from GENODIVE were shown by new numbers with thick circules. Un-genotyped samples are shown by "x". Genets that consisted of at least two ramets are shown by grey shades.

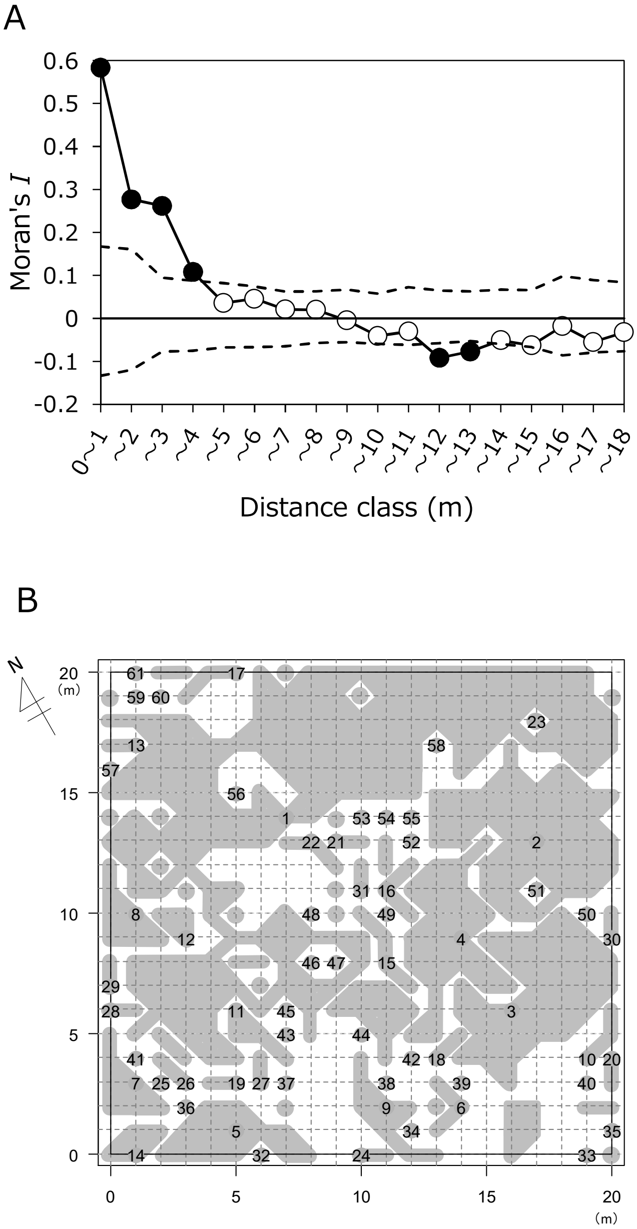

Figure S2 Spatial dependency of genet distribution based on genetic distances between genets (A, B). Spatial autocorrelation represented by a correlogram based on the genetic distances between genets (A). For the genets with multiple ramets, we chose a single ramet for each genet, the nearest one to the average coordinates of all ramets from the genet (B). In (A), Solid lines and circles represent Moran's *I* at different distances. Dotted lines represent 95% confidence limits in the null model based on 1,000 permutation tests. The filled circles represent significant deviations from the null model ($p < 0.05$). In (B), different numbers represent different genets, which correspond to those in **Fig. 4A**. Genets that consisted of at least two ramets are represented by grey-shaded areas.

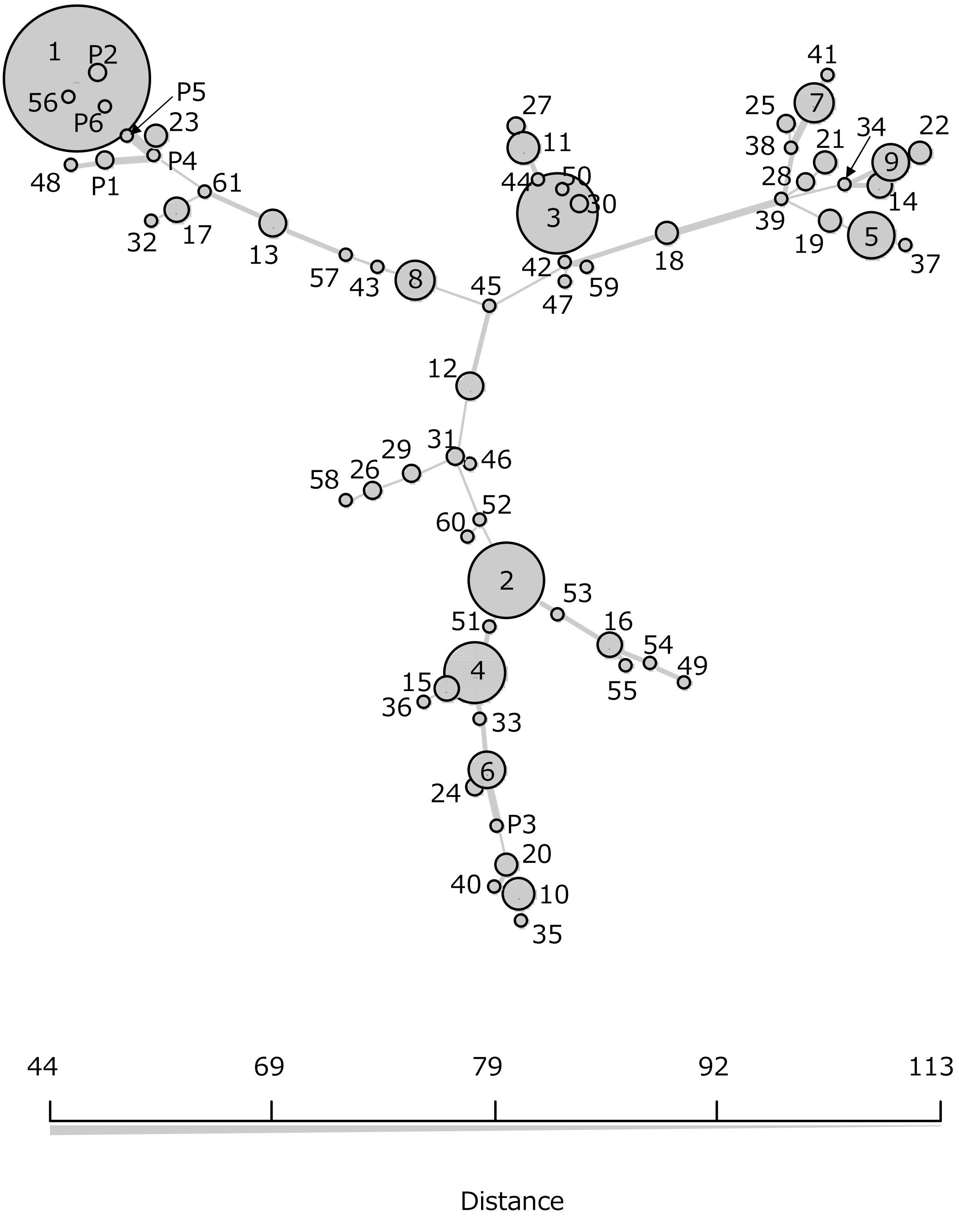

Figure S3 Minimum spanning network (MSN) of genets within the main plot drawn by an R package, *poppr*. Each node represents each genet. The size of a node is proportional to the number of ramets assigned to each genet. Numbers represent genets shown in **Fig. 4A** except for P1-P6, which were additionally assigned by *poppr* (**Figure S1C**). Connections with relative higher relatedness are indicated by width of lines. The position of the nodes and edge length are arbitrary.

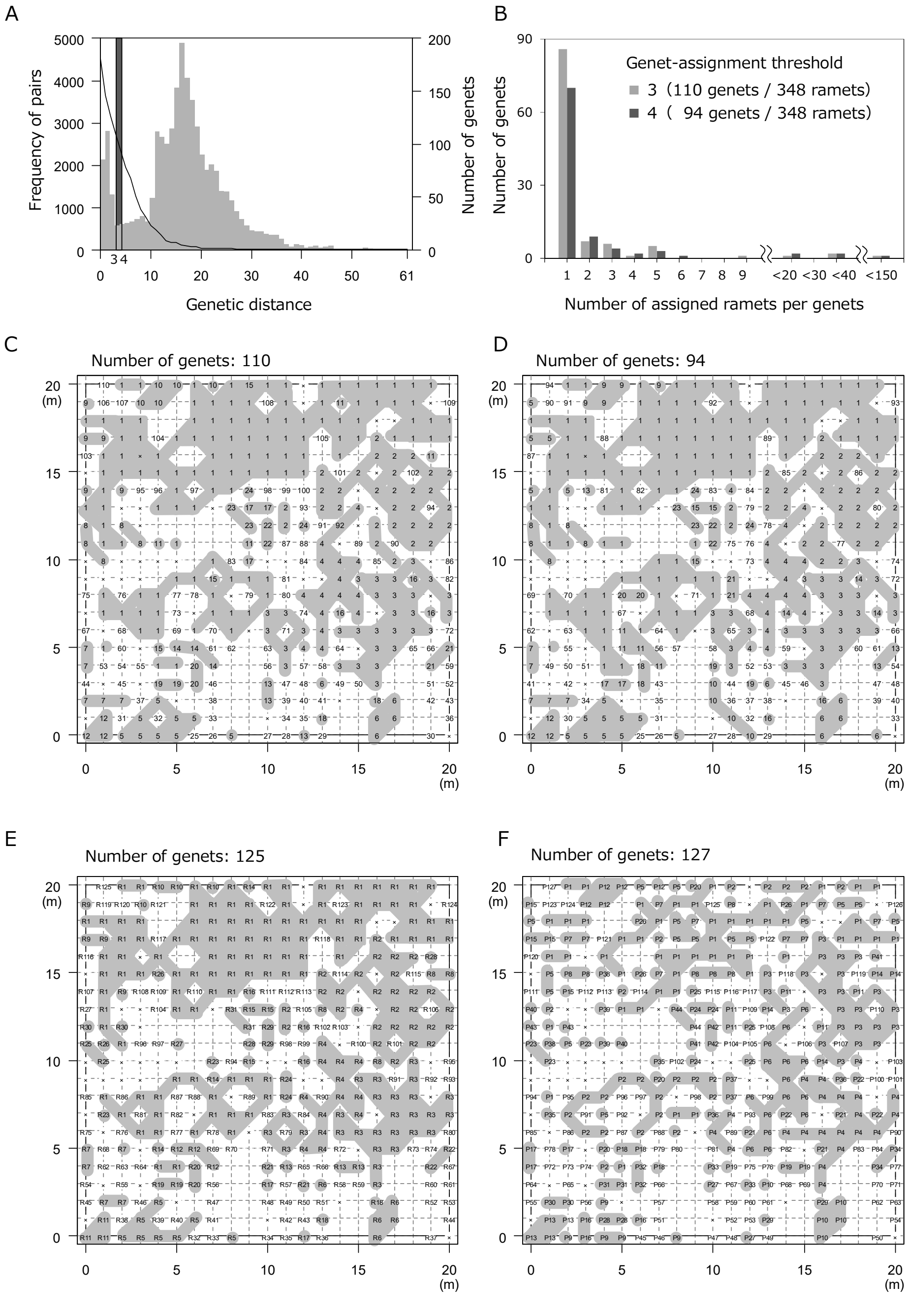

Figure S4 Genet assignment of ramets and genet structure of the main plot determined by the SSR analysis (A-F). Frequency distribution of pairwise genetic distances between ramet samples (A), frequency distribution of the number of ramets per genets at two genet assignment thresholds (genetic distance = 3 and 4, B), and spatial distribution of genets determined by GENODIVE (C and D), RClone (E) and *poppr* (F) are listed. In (A), genetic distances were calculated using stepwise allele model. Solid lines represent transition of the number of genets along with threshold genetic distance.

A dark grey area represents the threshold of genetic distance applied for genet assignment (ranged from 3 to 4). In (C-F), different number represents different genets. Genets were ranked by the number of assigned ramets in the samples. Un-genotyped samples are shown by "x". Genets that consisted of at least two ramets are shown by grey shaded areas.

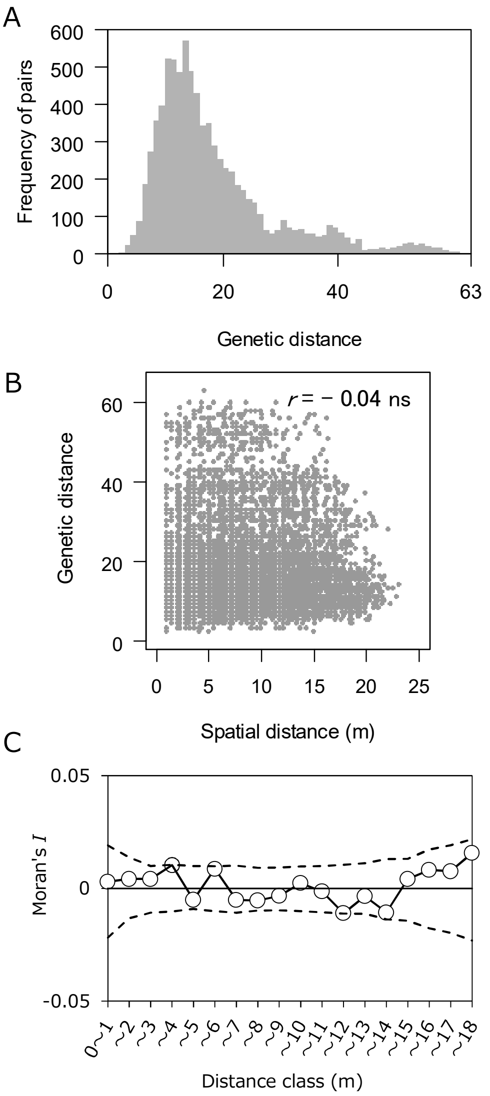

Figure S5 Genetic distance between ramets in RAD-seq within the largest genet (G1) and its spatial dependency (A-C). Frequency distribution of pairwise genetic distances between ramets (A), a scatter plot between genetic and spatial distances (B), and a correlogram in the spatial autocorrelation analysis (C) are listed. In (A), genetic distances were calculated using infinity allele model. In (B), correlation coefficient (*r*) is listed, and *ns* indicate non-significance in the Mantel test at $p < 0.05$. In (C), solid lines and circles represent Moran's *I* at different distances. Dotted lines represent 95% confidence limits in the null model based on 1,000 permutation tests. The filled circles represent significant ($p < 0.05$) deviations from the null model.

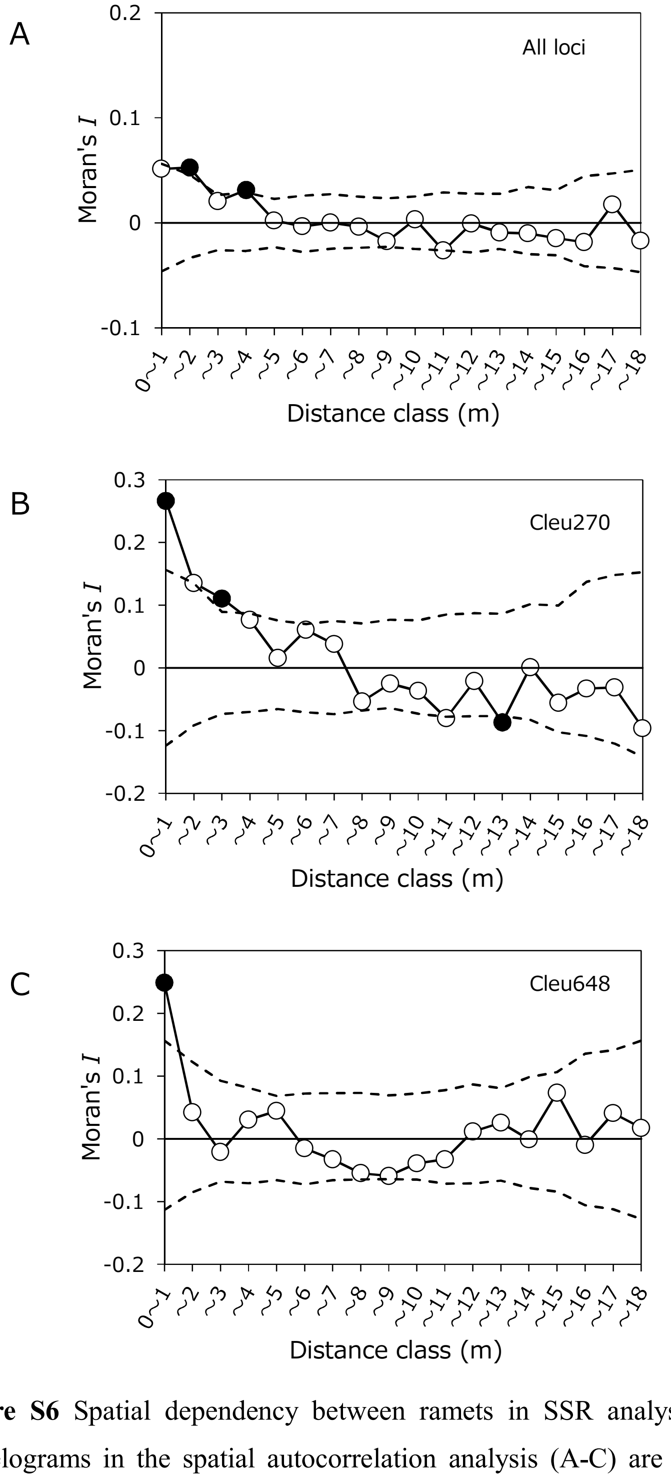

Figure S6 Spatial dependency between ramets in SSR analyses within the largest genet (G1). Correlograms in the spatial autocorrelation analysis (A-C) are listed. In (A), autocorrelation was estimated using all 13 SSR loci. In (B) and (C), autocorrelation was estimated using a single SSR locus; Cleu270 and Cleu648, respectively. Solid lines and circles represent Moran's *I* at different distances. Dotted lines represent 95% confidence limits in the null model based on 1,000 permutation tests. The filled circles represent significant ($p < 0.05$) deviations from the null model.

Supporting Information Tables

Table S1 Overview of the genet assignment procedure from the RAD-seq data.

Steps	Analyses	Used tools	Input data	Output data
Step1	Filtering of quality	Trimmomatic	Raw reads (Fastq file)	Trimmed reads
Step2	Mapping (Mapped to the draft genome sequence of *Cardamine leucantha*)	bowtie	Trimmed reads	Unique mapped reads
Step3	Building of contigs	Stacks	Unique mapped reads	Contigs and their sequences of each sample (total 179,265 contigs from 384 samples)
Step4	Choosing SNP loci	In-house script by R	Contigs and their sequences	Filtered SNP loci and genotypes [contigs with one SNP, diallelic, rare allele frequency in all samples ≥ 1 %, and obtained from ≥ 90 % of samples, dapth ≥ 10*; Samples with less than 90 % SNP loci were removed] (363 SNP loci of 372 sapmles)
Step5	Calculation of genetic distance	GENODIVE (RClone, *poppr*)	Filtered SNP loci and genotypes	Pairwise genetic distance
Step6	Setting of threshold for genet assignment	GENODIVE (RClone, *poppr*)	Pairwise genetic distance	Assigned genet

* We also used other criteria of the minimum depth, i.e. depth ≥ 5, ≥ 15, ≥ 20, and ≥ 30. The number of SNP loci, and estimated number of genets, clonal diversity, genet size inequalities, and genetic diversity were shown for the different criteria (see **Table S2**).

Table S2 Effects of the minimum depth in the filtering of SNP loci on the results in the RAD-seq analysis. Numbers of analyzed ramets (*N*), number of SNPs, the threshold genetic distance for genet assignment, number of assigned genets (*G*), clonal diversity (*G*/*N* and Simpson's *D*), and genet size inequalities are listed. *N* represents the number of samples remained after step 4 in **Table S1**. Depth ≥ 10 was used for the analyses in this study.

Depth	Number of ramets (*N*)	Number of SNPs	Threshold genetic distance		Number of genets (*G*)	Clonal diversity		Genet size inequalities
			Absolute	Relative percentages of max genetic distance (%)		*G* / *N*	Simpson's *D*	Gini coefficients
≥5	369	621	96	38.9	56	0.15	0.85	0.71
≥10	372	363	43	27.7	61	0.16	0.86	0.72
≥15	372	217	31	33.3	58	0.16	0.85	0.72
≥20	373	138	16	25.0	58	0.16	0.85	0.72
≥30	373	69	5	14.7	59	0.16	0.85	0.72

Table S3 The genetic variation within the top eight largest genets in the RAD-seq and SSR analysis. Numbers of analyzed ramets, number of polymorphic loci, average number of genotypes, and average number of alleles are listed.

Genets	RAD-seq (Total number of SNP loci : 363)				SSR (Total number of loci: 13)			
	Number of ramets	Number of polymorphic loci	Average number of genotypes	Average number of alleles	Number of ramets	Number of polymorphic loci	Average number of genotypes	Average number of alleles
G1	128	100	1.35	1.29	118	11	3.00	2.77
G2	38	32	1.11	1.19	31	6	1.85	1.92
G3	34	39	1.12	1.20	34	12	2.00	2.00
G4	22	35	1.12	1.18	18	6	1.77	2.00
G5	13	38	1.11	1.22	12	12	2.15	2.00
G6	9	46	1.19	1.31	6	7	1.69	1.77
G7	9	9	1.02	1.21	7	9	2.00	2.15
G8	9	6	1.02	1.20	5	3	1.31	1.62

*Here, "Genets" indicates that genets which were assigned using RAD-seq data by GENODIVE.

*The number of ramets analyzed by RAD-seq and SSR was different because some ramets were failed to be genotyped by SSR.
